# A high-resolution 3D epigenomic map reveals insights into the creation of the prostate cancer transcriptome

**DOI:** 10.1038/s41467-019-12079-8

**Published:** 2019-09-12

**Authors:** Suhn Kyong Rhie, Andrew A. Perez, Fides D. Lay, Shannon Schreiner, Jiani Shi, Jenevieve Polin, Peggy J. Farnham

**Affiliations:** Department of Biochemistry and Molecular Medicine and the Norris Comprehensive Cancer Center, Keck School of Medicine, University of Southern California, Los Angeles, CA 90089 USA

**Keywords:** Cancer genomics, Epigenomics, Gene regulation, Chromatin structure

## Abstract

To better understand the impact of chromatin structure on regulation of the prostate cancer transcriptome, we develop high-resolution chromatin interaction maps in normal and prostate cancer cells using in situ Hi-C. By combining the in situ Hi-C data with active and repressive histone marks, CTCF binding sites, nucleosome-depleted regions, and transcriptome profiling, we identify topologically associating domains (TADs) that change in size and epigenetic states between normal and prostate cancer cells. Moreover, we identify normal and prostate cancer-specific enhancer-promoter loops and involved transcription factors. For example, we show that FOXA1 is enriched in prostate cancer-specific enhancer-promoter loop anchors. We also find that the chromatin structure surrounding the androgen receptor (AR) locus is altered in the prostate cancer cells with many cancer-specific enhancer-promoter loops. This creation of 3D epigenomic maps enables a better understanding of prostate cancer biology and mechanisms of gene regulation.

## Abstract (web summary)

In prostate cancer, chromatin structure can impact the transcriptome. Here, the authors develop high resolution chromatin interaction maps in prostate cancer cells using in situ Hi-C, revealing prostate cancer-specific TADs and enhancer-promoter loops surrounding the androgen receptor (AR) locus.

## Introduction

Prostate cancer is the leading cause of new cancer cases and the second most common cancer in men and the fourth most common tumor type worldwide. Multiple genetic and environmental factors including genetic susceptibility, family history, age, and race contribute to prostate cancer risk^1–4^. Unlike normal prostate cells, prostate cancer cells have greatly dysregulated transcriptome profiles, causing uncontrolled cell proliferation^2,3^. Clearly, new insights into the mechanisms by which the prostate cancer transcriptome is controlled would provide valuable information for diagnosis and treatment.

The human genome is organized in three-dimensional (3D) space in the nucleus into active and inactive compartments and regions of high within contact frequency called topologically associating domains (TADs). Protein–protein interactions involving cohesin complexes and CTCF mediate long-range chromatin looping. The general activity of a TAD can be determined by its epigenomic profile (i.e. which types of modified histones are bound), being classified as either open (active) or closed (repressive)^5,6^. Although previous studies have found that the boundaries of most TADs are conserved across cell types^7,8^, the epigenomic profiles (and thus the chromatin states) of a TAD can differ by cell type. Also, as cells change from one cell type to another, certain TADs transition between active and inactive chromatin states or vice versa. The epigenetic state of a TAD can influence the general expression level of genes within a given TAD. Moreover, genes are most often regulated by enhancers located within the same TAD. Alteration of the chromatin loops that occur between pairs of regulatory elements bound by CTCF can bring together (or split apart) a promoter from a distal regulatory element, causing changes in gene expression. Thus, the transcriptome can be affected by changes in TAD boundaries. Finally, cancer-specific changes in the activity of regulatory elements such as enhancers (caused by the gain or loss of TFs) can also cause alterations in expression of specific genes due to changes in long-range enhancer–promoter loops^9,10^.

To study these chromatin interactions, various chromatin conformation capture assays have been developed. Hi-C, a genome-wide chromatin conformation capture assay, has been particularly useful for mapping the very large structural TADs^11^. By performing in situ Hi-C and increasing the sequencing depth of the in situ Hi-C libraries, high-resolution intra-TAD chromatin interactions between regulatory elements such as promoters and enhancers can be found^5,12,13^. Moreover, the epigenetic state of the interacting regions can be characterized using ChIP-seq of specifically modified histones, which mark regulatory elements. TFs that are involved in the chromatin interactions can be inferred from motif analyses using methods such as DNase-seq, ATAC-seq, or NOMe-seq (Nucleosome Occupancy and Methylome sequencing), which define nucleosome-depleted regions (NDRs) that correspond to TF-binding platforms, and the binding sites of TFs can be further identified by ChIP-seq.

To better understand the mechanisms by which the prostate cancer transcriptome is controlled, we have used a combination of these methods in normal prostate and prostate cancer cells to (a) map TAD boundaries, (b) investigate the epigenetic state of the TADs, and (c) identify and characterize normal- and cancer-specific enhancer–promoter loops. We performed high-resolution in situ Hi-C in normal prostate cells and prostate cancer cells, sequencing ~1 billion reads per cell line. We also performed ChIP-seq for modified histones and CTCF, NOMe-seq, and RNA-seq in normal and prostate cancer cells. This characterization of TADs and chromatin loops provides more detailed epigenetic mechanisms of prostate tumorigenesis.

## Results

### Cancer-specific TADs that lead to transcriptome changes

**Fig. 1:**
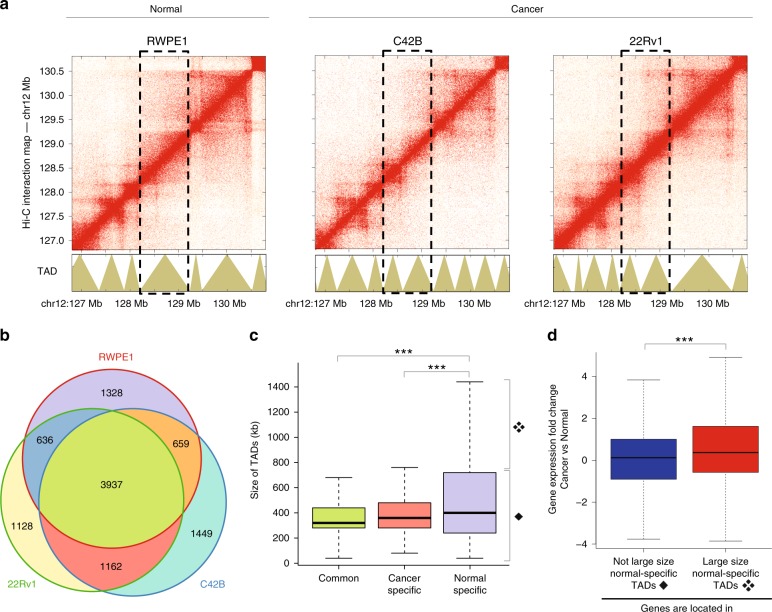
Changes in TAD boundaries leads to alterations in prostate cancer transcriptome. **a** In situ Hi-C chromatin interaction maps of the region of chromosome 12q24 in normal (RWPE1) and prostate cancer (C42B, 22Rv1) cells. Red indicates more frequent interactions and white indicates no interactions. TADs identified using the TopDom program^14^ are shown at the bottom. Dashed lines indicate the position of a single TAD in RWPE1 cells, which forms two TADs in the cancer cells. **b** A Venn diagram showing the overlap of TADs found in the three cell lines. **c** Shown is the size of common (*n* = 3937), normal-specific (*n* = 1328), and cancer-specific (*n* = 1162) TADs. **d** Expression fold change between cancer (C42B) and normal (RWPE1) cells of genes that are located in large size normal-specific TADs (red, see the symbol ❖ to the right side of graph in **c**) and not large size normal-specific TADs (blue, solid diamond ♦) (Wilcoxon rank sum test, *adj. *p* value < 0.05, **adj. *p* value < 0.01, ***adj. *p* value < 0.001)

Using in situ Hi-C with a 4-cutter restriction enzyme MboI, we created high-resolution chromatin contact interaction profiles (~1 billion reads per cell line, please see Methods for detailed sequencing and replicate information) in normal prostate (RWPE1) and prostate cancer (C42B and 22Rv1) cells (Fig. 1a; Supplementary Data 1), identifying 6565, 7205, and 6861 TADs in RWPE1, C42B, and 22Rv1, respectively (Supplementary Fig. 1, Supplementary Data 2) using the TopDom software^14^. More than 80% of TADs are reproducible between replicates (Supplementary Fig. 2). Among all three cell lines, we found that 3937 (~60%) of the TADs share boundaries (Fig. 1b); these common TADs have an average size of ~350 kb (Fig. 1c). We also identified 1328 TADs that were detected in normal RWPE1 cells but not in either cancer cell line (classified as normal-specific TADs, average size ~600 kb) and 1162 TADs that were detected in both cancer cell lines but not in the normal cells (classified as cancer-specific TADs, average size ~400 kb). Interestingly, the normal-specific TADs were generally larger than the cancer-specific TADs (adj. *p* value < 2.26e-05, Wilcoxon rank sum test); see Fig. 1a and Supplementary Fig. 3 for examples, Fig. 1c and Supplementary Fig. 4 for a genome-wide analysis of TAD size.

Moreover, we found that the size change of a TAD can be related to gene expression changes. For example, in normal cells, there is one large TAD at chromosome 15q12 containing the *GABRB3* gene, which is not expressed in RWPE1. However, in the cancer cells, at the same genomic region at chromosome 15q12, the one large TAD is split into two smaller-size TADs and the *GABRB3* gene is expressed (Supplementary Fig. 5). By comparing TAD sizes between normal and cancer cells genome-wide (adj. *p* value < 0.05, Wilcoxon rank sum test), we identified ~520 large size TADs in normal cells that correspond to ~850 smaller TADs in cancer cells. Interestingly, we found that in these altered TADs, relatively more genes showed increased expression in cancer cells than in normal cells (*p* value < 8.93e-09, Wilcoxon rank-sum test) (Fig. 1d). Among ~1800 genes found in these altered TADs, ~500 genes showed significantly higher expression in cancer cells than in normal cells (fold change > 2, adj. *p* value < 0.05) (Supplementary Data 3); the number of these upregulated genes in cancer cells was more than two times the number of downregulated genes in these altered TADs. We also found that common TADs, which are smallest in size (Fig. 1c), have relatively more genes than cell-type-specific TADs (gene-enriched TADs: common vs normal-specific adj. *p* value < 1.26e-02, common vs cancer-specific adj. *p* value < 8.93e-04, Fisher’s exact test) (Supplementary Fig. 6). Many of the genes in the smaller TADs are more transcriptionally active, suggesting that perhaps the smaller TAD (e.g. cancer-specific smaller TADs) insulates the gene from repressive elements (e.g. normal-specific larger TADs).

### Common TADs that can change chromatin states

**Fig. 2:**
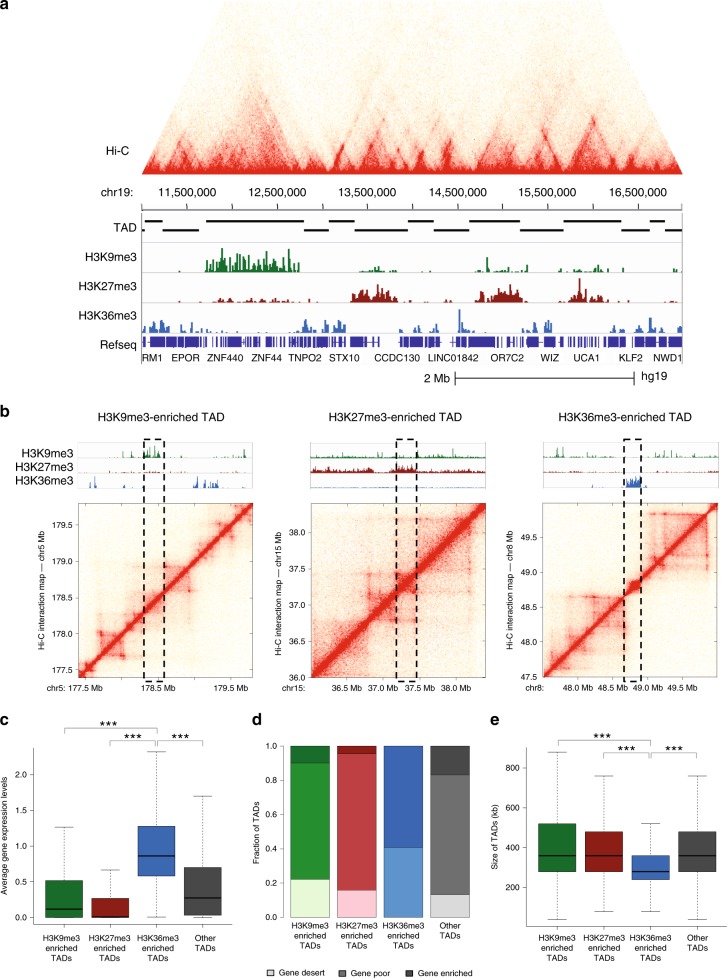
Identification of histone mark-enriched TAD subgroups. **a** An example of an in situ Hi-C chromatin interaction map with histone-mark ChIP-seq tracks of a region of chromosome 19p13 in C42B prostate cancer cells. **b** Shown are in situ Hi-C chromatin interaction maps for a H3K9me3-, H3K27me- and H3K36me3-enriched TAD in C42B prostate cancer cells. Histone-mark ChIP-seq tracks from C42B prostate cancer cells are also shown. **c** Average expression levels of genes found in histone-mark-enriched TADs and TADs without those histone marks (other). **d** Fraction of gene desert (light), gene poor (mid), and gene enriched (dark) TADs for the histone mark-enriched TAD subgroups and other TADs. **e** Shown is the size of histone mark-enriched TADs and other TADs (Wilcoxon rank sum test, *adj. *p* value < 0.05, **adj. *p* value < 0.01, ***adj. *p* value < 0.001)

The above analyses identified TADs that have different boundaries in normal and cancer cells and correlated these boundary changes with changes in gene expression. However, they did not provide information concerning the overall nature of the chromatin state of the TADs or how the epigenetic state may influence the expression level of the genes within the TADs. Therefore, we further characterized the TADs by performing ChIP-seq in normal and prostate cancer cells with antibodies that demarcate active and inactive regions (Fig. 2a, Supplementary Data 2). We used the H3K9me3 heterochromatic histone mark to annotate heterochromatic TADs, the H3K27me3 repressive histone mark to annotate repressed TADs, and the transcription elongation histone mark H3K36me3 to annotate active TADs. Examples of a heterochromatic, a repressed, and an active TAD are shown in Fig. 2b. H3K36me3 is a mark that is only present within gene bodies of expressed genes. As expected, the average gene expression level is higher in H3K36me3-enriched TADs than other subgroups of TADs (Fig. 2c). The H3K36me3-enriched TADs also display the highest gene density and are the smallest in size (Fig. 2d, e). In contrast, the H3K9me3-enriched TADs have the lowest gene density (i.e. the highest percentage of gene deserts) and are the largest TADs, which correlate well with the fact that this mark is known to cover large heterochromatic regions (Fig. 2d, e). A similar pattern of the size, gene density, and gene expression levels for the epigenetic state-specific TADs are identified across cell types (Supplementary Fig. 7).

**Fig. 3:**
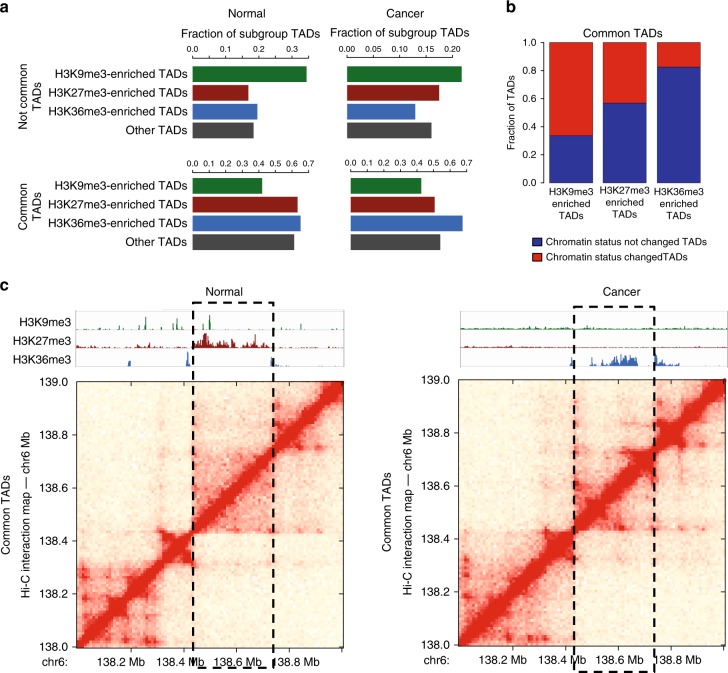
Identification of TADs that change epigenetic states in prostate cancer cells. **a** Top: fraction of histone mark-enriched TAD subgroups in normal-specific TADs (RWPE1) and cancer-specific (C42B) TADs. Bottom: fraction of histone mark-enriched TAD subgroups in common TADs (left: normal (RWPE1), right: cancer (C42B)). **b** Shown is the fraction of common TADs whose chromatin status changed (red) and did not change for each histone mark-enriched TAD subgroup (blue); the initial epigenetic status of each TAD was from normal (RWPE1) cells. **c** Shown is an example of a common TAD located at chromosome 6q23 that changed epigenetic status between normal and prostate cancer cells. Dashed lines indicate the same TAD boundaries in both the normal and cancer cells

Having characterized the epigenetic states of all TADs in RWPE1, C42B, and 22Rv1 cells, we next specifically examined the epigenetic states and expression levels within the sets of common, normal-specific, and cancer-specific TADs. We found that normal-specific TADs have a higher percentage of H3K9me3-enriched TADs than either the cancer-specific TADs or the common TADs (analyzed in either normal or cancer cells) (Fig. 3a). As shown in Fig. 1c, normal-specific TADs also tend to be larger than cancer-specific TADs. This suggests that perhaps a subset of large, heterochromatic TADs that form in normal prostate cells are split into smaller, active TADs during neoplastic transformation, providing support for the hypothesis proposed above that larger normal-specific TADs may contain repressive elements that keep gene expression low.

In addition, we examined the common TADs, which share boundaries among normal and cancer cells. We found that 66.5% of H3K9me3-enriched common TADs had different epigenetic state in the normal vs cancer cells, with lesser percentages of the H3K27me3 TADs changing state (43.2%) and very few H3K36me3 TADs changing state (17.5%) (Fig. 3b) (H3K9me3 vs H3K27me3: adj. *p* value < 5.93e-05, H3K9me3 vs H3K36me3: adj. *p* value < 7.44e-25, H3K27me3 vs H3K36me3: adj. *p* value < 1.80e-13, Fisher’s exact test). An example of a common TAD found at chromosome 6q23 that has a different epigenetic state in normal cells (H3K27me3) and cancer cells (H3K36me3) is shown in Fig. 3c. Overall, we identified ~2000 genes that reside in the sets of changed epigenetic state TADs. Of these, >40% of genes changed expression level significantly (absolute fold change > 2, adj. *p* value < 0.05) (Supplementary Data 4); there was ~50% increase in the number of differentially expressed genes in the changed epigenetic state TADs compared to the non-changed state TADs.

### Enhancer–promoter loops in prostate cancer cells

**Fig. 4:**
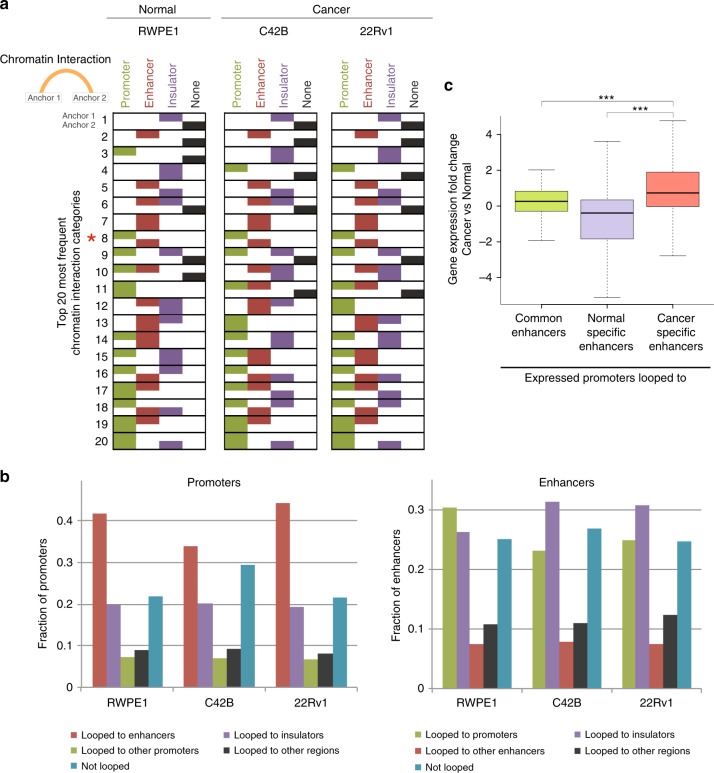
Identification of enhancer–promoter loops. **a** Shown are the top 20 most frequent chromatin interaction categories for RWPE1, C42B, and 22Rv1, in rank order with the most frequent category at the top and the 20th most frequent category at the bottom. Chromatin interaction categories have two anchors; for each loop, the type of regulatory element at anchor 1 is shown on the top half of the rectangle and the type of regulatory element at anchor 2 is shown on the bottom half of the rectangle. Therefore, each rectangle indicates if anchors 1 and/or 2 contain a promoter (green), enhancer (red), insulator (purple), or none of these regulatory elements (black). The enhancer–promoter chromatin interaction category (which is ranked as the eighth most frequent category in each cell line) is indicated with a red asterisk. **b** Shown for RWPE1, C42B, and 22RV1 is the fraction of active promoters (left) that loop to enhancers, insulators, other promoters, other genomic regions that are not marked as active regulatory elements or are not looped, and the fraction of active enhancers (right) that loop to promoters, insulators, other enhancers, other genomic regions that are not marked as active regulatory elements or are not looped. **c** Expression fold change between cancer (C42B) and normal (RWPE1) cells of genes that have promoters looped to common, normal-specific, and cancer-specific enhancers (Wilcoxon rank sum test, *adj. *p* value < 0.05, **adj. *p* value < 0.01, ***adj. *p* value < 0.001)

To characterize high-resolution chromatin contact loops, we identified intra-chromosomal significant loops (using a range of 50 kb to 10 Mb) in normal and prostate cancer cells using the Fit-HiC program^15^. We focused on regulatory loops, identifying chromatin anchors that overlapped with active promoters (defined as ±2 kb of TSSs of expressed genes, as determined using RNA-seq data), active enhancers (defined as H3K27ac ChIP-seq peaks that are not near a TSS), and CTCF-binding sites (defined as CTCF ChIP-seq peaks that are not overlapping with the sets of promoters or enhancers). Using a *q*-value cutoff of 0.05, we found that ~70% of the promoter and enhancer regions and ~90% of the CTCF peaks overlapped with a chromatin interaction anchor. In the top 20 most frequent chromatin interaction categories, most loops had a CTCF site at one or both anchor regions, providing evidence in support of the robustness of the analysis methods. We also identified sets of loops involving both enhancers and promoters (Fig. 4a). The loop anchor enrichment at these regulatory elements was statistically significant compared to random genomic regions with same size of loop anchors (empirical *p* value < 0.01). It is important to note that although ~70% of the promoters are involved in loops, less than 50% of the promoters looped to enhancers (Fig. 4b). Similarly, although ~70% of enhancers are involved in loops, less than 30% of the enhancers looped to promoters (Fig. 4b). We next focused on the specific subset of enhancer–promoter loops. We identified 7948 loops found in the normal RWPE1 cells but not in either cancer cell line (normal-specific enhancer–promoter loops), 1098 loops found in both cancer cell lines but not in the normal cells (cancer-specific enhancer–promoter loops), and 426 loops found in all cells (common enhancer–promoter loops) (Supplementary Data 5), using a *q*-value cutoff of 0.05 (see Methods). We then further subdivided genes having the enhancer–promoter loops into genes having loops from a promoter to a common enhancer vs from a promoter to a normal-specific or cancer-specific enhancer. Interestingly, the genes regulated by promoters looped to cancer-specific enhancers show higher gene expression in the cancer cells, as compared to genes regulated by promoters looped to normal-specific or common enhancers (cancer-specific vs normal-specific adj. *p* value < 2.26e-170, cancer-specific vs common adj. *p* value < 2.11e-06) (Fig. 4c), suggesting that the cancer-specific loops contribute to an increase in gene expression.

### TFs at cell-type-specific enhancers that loop to promoters

**Fig. 5:**
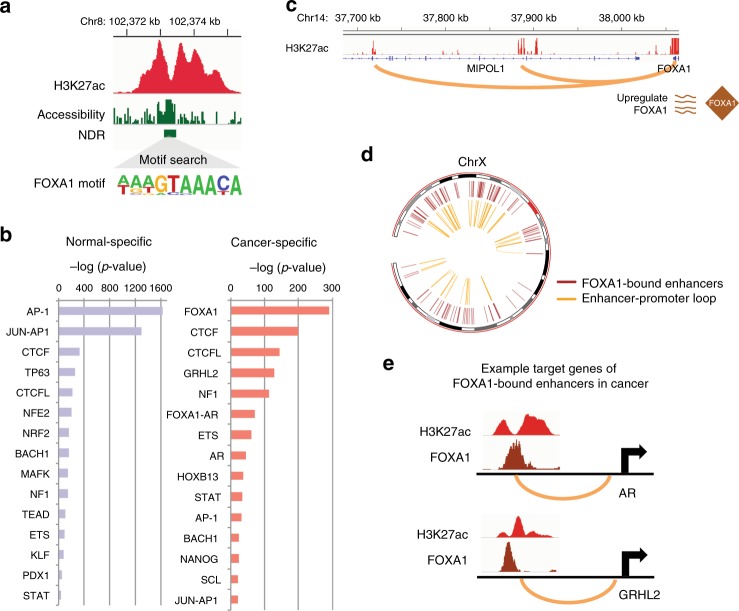
TFs enriched at cell-type-specific enhancers that loop to promoters. **a** Shown is an example of a cancer-specific enhancer at chromosome 8q22.3 that has a FOXA1 motif within the NDR. **b** Shown are the top most frequent TF-binding motifs found in NDRs within enhancers that loop to promoters. Left: Normal-specific enhancer NDRs. Right: Cancer-specific enhancer NDRs. **c** A genome browser snapshot of the H3K27ac ChIP-seq track and enhancer–promoter loops identified by in situ Hi-C for the FOXA1 promoter in prostate cancer cells (C42B). **d** An example of Circos plots showing FOXA1binding sites at C42B enhancers in chromosome X (outer circle) and the enhancer–promoter loops of FOXA1-bound enhancers in C42B (inner circle). **e** Example target genes (top: AR; bottom: GRHL2) of FOXA1-bound enhancers in prostate cancer cells. Shown are the ChIP-seq tracks for H3K27ac and FOXA1 in C42B cells

Transcription factors (TFs) are key regulators that can drive changes in transcription of numerous genes along pathways by binding to DNA regulatory elements. Cell-type-specific TFs^2,16^ can, upon binding to regulatory elements and recruitment of HATs, cause the creation of a cell-type-distinguishing H3K27ac epigenomic (enhancer) profile. Also, chromatin interaction studies of different cell types have identified TFs that are specifically involved in looping^12,13,17^. Therefore, we next identified the TF-binding motifs that are found within the active enhancers that loop to promoters in normal (RWPE1) and prostate cancer (C42B) cells. H3K27ac ChIP-seq peaks are broad and cover many more nucleotides than the actual TF binding platforms (Fig. 5a). However, NOMe-seq can identify the NDRs within each enhancer, narrowing the motif search window to ~150 bp regions corresponding to TF landing platforms within the broad H3K27ac ChIP-seq peaks^18^. As an example, an NDR within a large H3K27ac peak (~3 kb) located at chromosome 8q22.3 is identified by NOMe-seq; motif search on this NDR identified a FOXA1 motif (Fig. 5a). Therefore, we first used NOMe-seq to find NDRs (Supplementary Data 6) and extract the genomic locations of the TF-binding platforms from the H3K27ac peaks genome-wide in RWPE1 and C42B cells. We next analyzed the subset of NDRs found in normal-specific enhancers that we identified to be involved in enhancer–promoter loops, identifying motifs for TFs such as AP-1, TP63, and NFE2. On the contrary, NDRs in prostate cancer-specific enhancers involved in enhancer–promoter loops are enriched for motifs for TFs such as FOXA1, GRHL2, ETS, and AR (Fig. 5b). Both sets of NDRs had CTCF and CTCFL motifs, which is consistent with the fact that these enhancers were selected as the subset that were identified by Hi-C data as being involved in looping.

We found that FOXA1 is the TF motif most frequently enriched at enhancers in prostate cancer-specific enhancer–promoter loops (Fig. 5b). RNA-seq analysis indicates that FOXA1 is upregulated in prostate cancer cells (fold change > 498.3, adj. *p* value < 1.5e-07) (Supplementary Data 7). According to the Cancer Genome Atlas (TCGA) gene expression datasets, FOXA1 is also expressed higher in prostate tumor tissue samples than normal tissue samples (*p* value < 4.24e-03, Student’s *t*-test) (Supplementary Fig. 8). Using our in situ Hi-C and epigenomic data, we identified two strong cancer-specific H3K27ac peaks located at ~350 and ~180 kb upstream of the transcription start site of *FOXA1* that are looped to the *FOXA1* promoter region in C42B prostate cancer cells (Fig. 5c); the interaction of each loop was more frequent in cancer cells compared to normal cells. One mechanism by which the transcriptome can be altered in cancer cells is by the action of upregulated TFs. Therefore, we next investigated the role of FOXA1 in regulating the cancer transcriptome. Using a previously published FOXA1 ChIP-seq dataset^19^, we identified ~7000 FOXA1-binding sites in enhancers. We further narrowed the FOXA1-bound enhancers to those we identified to be involved in enhancer–promoter loops. We found that 31% (1113/3591) of the cancer-specific enhancers that are involved in enhancer–promoter loops in C42B prostate cancer cells are bound by FOXA1, suggesting that FOXA1 is a major regulator of the cancer transcriptome (see Fig. 5d which shows enhancers and loops for an example chromosome, chrX). Thus, enhancer–promoter loops not only lead to increased FOXA1 expression, but the upregulated FOXA1 protein then also contributes to the cancer transcriptome by mediating many additional enhancer–promoter loops which lead to increased gene expression. Inspection of the set of genes (Supplementary Data 8) whose promoter is looped to FOXA1-bound enhancers revealed that genes involved in cell cycle (e.g. *CDK4, CDC23, MYC*) as well as the androgen receptor (*AR*) and androgen-responsive genes (e.g. *GRHL2*) are target genes of FOXA1-bound enhancers (Fig. 5e).

### The chromatin structure surrounding the AR locus

**Fig. 6:**
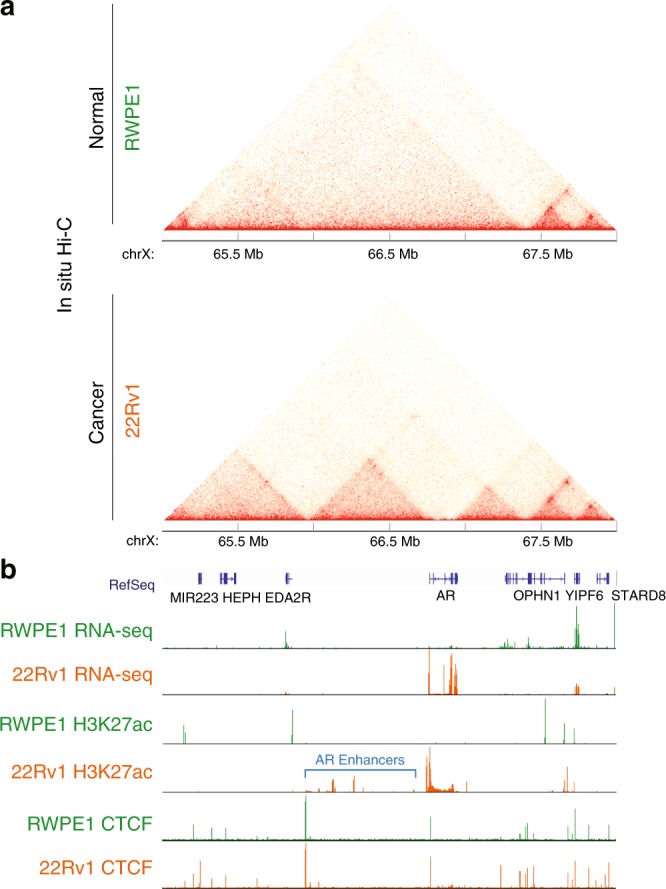
Chromatin structure changes surrounding the AR locus in prostate cancer cells. **a** In situ Hi-C chromatin interaction maps of normal (RWPE1) and prostate cancer (22Rv1) cells for the region surrounding the AR locus. **b** RNA-seq, H3K27ac, and CTCF ChIP-seq tracks of normal (RWPE1) and prostate cancer (22Rv1) cells for the AR locus. The potential AR enhancers within the TAD where AR promoter resides are shown in a blue bracket

To further study enhancer–promoter loops related to the prostate cancer transcriptome, we selected the most frequent prostate cancer-specific enhancer–promoter loops by performing Fisher’s exact test, comparing the loops in normal vs cancer cells. Then, we identified the subset of these interactions, which have an enhancer that loops to a promoter of a gene that is statistically significantly higher expressed in prostate cancer cells than in normal cells (Supplementary Data 5, 9). Among those, we found that enhancer–promoter loops for the *AR* gene are specific to the prostate cancer cells. In situ Hi-C maps confirm that the enhancer–promoter interactions are more frequently detected in 22Rv1 prostate cancer cells than in RWPE1 normal cells; there are also cancer-specific smaller-size TADs near the *AR* gene (Fig. 6a). Accordingly, H3K27ac ChIP-seq profiles show that multiple enhancers found within a TAD where the promoter of *AR* gene is located are active in 22Rv1 prostate cancer cells, but not in RWPE1 normal cells (Fig. 6b). More chromatin contact frequencies in cancer cells may facilitate to upregulate *AR* expression in 22Rv1 prostate cancer cells compared to RWPE1 normal cells (fold change >454.8, adj. *p* value < 3.1e-07) (Supplementary Data 7). We also found that CTCF-binding sites are located at the boundaries of the prostate cancer-specific TADs. However, unlike enhancer and chromatin contact profiles, which show dramatic differences between normal and cancer cells, the CTCF profiles were not that different.

## Discussion

In this study, we investigated mechanisms of gene regulation in prostate cancer related to chromatin structure changes using three different approaches. First, we identified more than a thousand TADs that have altered boundaries between normal and prostate cancer cells and then identified a set of genes whose expression is increased in the cancer-specific TADs (Supplementary Data 3). Second, we showed that >20% of the common TADs (i.e. the TADs having similar boundaries in normal and cancer cells) changed epigenetic status and then identified genes located in the common TADs that showed changes in expression correlating with an epigenetic status change from an inactive to an active TAD (Supplementary Data 4). Third, by intersecting regulatory elements with chromatin interaction loops, we identified genes regulated by prostate cancer-specific enhancer-promoter loops (Supplementary Data 9).

Using a dilution Hi-C method, a previous study^20^ reported 300 to 1000 TADs that have an average size of >2 Mb in prostate cells, which included a set of cancer-specific TADs linked to genetic alterations such as copy number variation. In comparison, we have used the in situ Hi-C method, which can map chromatin structure at a much higher resolution^5^, allowing us to better define the 3D architecture of the prostate cancer epigenome. We generated high-resolution chromatin interaction maps by performing in situ Hi-C using a 4-cutter restriction enzyme with deep sequencing, along with ChIP-seq experiments that profiled active and inactive chromatin states genome-wide in normal and prostate cancer cells. Using the TopDom program^14^, which can call TADs that are relatively robust to resolution and normalization among many TAD prediction tools^21,22^, we identified ~6500 TADs per cell line. Analysis of generating artificial TADs by random binning in the genome supports that TAD boundaries are not randomly generated but potential functional units (H3K36me3-enriched TADs, empirical *p* value < 0.01).

We identified more than a thousand cancer-specific TADs, which, in general, tend to be smaller than normal-specific TADs; the TAD size distribution of normal-specific TADs is very skewed with some TADs >3 Mb (Fig. 1c, Supplementary Figs. 3 and 4). Interestingly, many of the genes in these smaller cancer-specific TADs are more transcriptionally active (Supplementary Data 3), suggesting that the smaller TAD insulates the gene from distal repressive elements. Examples of genes that belong to this category are *TBX3* and *PRMT6*, which are known to be involved in prostate cancers^23,24^. The findings that transcriptionally active TADs (H3K36me3-enriched TADs) are smallest in size than other histone mark-enriched TADs (Fig. 2e), and that common TADs, which are smallest in size (Fig. 1c), have relatively more genes than cell-type-specific TADs (Supplementary Fig. 6) also support that the size of TADs is related to gene expression.

We also identified different epigenetic state-containing common TADs that harbor ~1000 differentially expressed genes between normal and prostate cancer cells (Supplementary Data 4). The epigenetic state change in a TAD may affect expression levels of many genes within one TAD. Examples of genes that belong to this category are CBX2, CBX4, and CBX8. These genes are located within a TAD that is repressed by H3K27me3 in normal prostate cell at chromosome 17q25.3. However, in prostate cancer cells, the expression levels of these genes are higher; no repressive marks are found in the TAD in cancer cells. CBX2 is reported as a potential therapeutic target in advanced prostate cancer as it is recurrently upregulated in metastatic Castrate Resistant Prostate Cancer (CRPC)^25^. CBX4 and CBX8 are also known to be involved in multiple cancer types^26–28^.

The high resolution of in situ Hi-C also allowed the identification of chromatin loops (in addition to the TADs). As anticipated, the loops we identified by in situ Hi-C are mostly found between pairs of CTCF/cohesin complexes, providing assurance that our experimental and analytical results are robust. Interestingly, only a small percentage of the loops we identified are between enhancers and promoters (even with the high depth of sequencing of the Hi-C libraries). It is possible that targeted sequencing methods such as promoter capture Hi-C^29^, Capture-C^30^, ChIA-PET^31^, or HiChIP^32^ may have detected more loops involved with active regulatory elements. However, it is clear that the enhancer–promoter loops that are detected by in situ Hi-C are not as frequent (i.e. robust) as the CTCF/cohesin loops, suggesting that any enhancer–promoter loops not detected in our studies are likely to be quite weak. The low interaction frequency of the enhancer–promoter loops may be due to the fact that, unlike CTCF which is stably bound to the chromatin, TFs that mediate these loops bind to DNA in a dynamic manner^33^.

To gain insight into the TFs that are bound at the strongest enhancer–promoter loops, we performed NOMe-seq to call NDRs and performed motif analyses using the nucleosome-depleted regions within the looped enhancers. We identified distinct TF motifs at enhancers that loop to promoters in normal vs cancer cells. For instance, normal-specific enhancers had motifs for AP-1, TP63, and NFE2, which are known to be enriched in open chromatin in several types of normal, differentiated cells^12,13,16^. On the contrary, prostate-cancer-specific enhancers had motifs for FOXA1, GRHL2, AR, and ETS. AR, FOXA1, and GRHL2 (a co-regulator of AR) are key TFs that promote prostate tumor progression^19,34^. ETS family TFs such as ERG, ETV1, and ETV4, are known prostate oncogenic regulators^3,35^. Using ChIP-seq data for the most enriched TF FOXA1, we also revealed target genes of prostate-cancer-specific enhancers. For example, *HOXC6* and *DLX1* genes, which are biomarkers and key players of prostate cancer development^2,36^ as well as genes involved cell cycle (*CDK4, CDC23, MYC*) and androgen signaling (*AR*, *GRHL2*) are found.

An example locus that includes cancer-specific enhancer–promoter loops is the *AR* locus (Fig. 6). 22Rv1 cells, which are CRPC cells, had new smaller TADs unlike RWPE1 normal prostate cells at the loci. 22Rv1 cells express an *AR* transcript variant 7 (AR-V7). AR-V7 contains the N-terminal transactivation and DNA-binding domains of AR, but lacks the ligand-binding domain that is present in full-length AR (AR-FL). The presence of AR-V7 is known to be associated with resistance to hormone therapy^37^. The identified enhancers involved in cancer-specific enhancer–promoter loops may have an effect on expression of *AR* gene, which includes *AR-V7* isoform. Others have shown that a regulatory element can be an oncogenic driver for advanced prostate cancer^38^. Moreover, as targeting AR splice variants have proved to be challenging^39^, further investigation of this AR-V7 enhancer will be beneficial for understanding the etiology of prostate cancer subtypes as well as potentially developing future clinical treatments for CRPC.

We found that CTCF-binding sites are located at the prostate cancer-specific TAD boundaries for AR loci both in normal and cancer cells (Fig. 6). We have previously shown that CTCF sites can play an important role in keeping gene expression levels low for nearby genes located in enhancer deserts. In our previous study, deleting CTCF-binding sites located at chromatin loop anchors of repressed regions resulted in high expression of the nearby genes, probably due to an enhancer adoption mechanism^40^. These findings support the model that chromatin interactions and structural changes can be key contributors for gene regulation in prostate cancer transcriptome. However, there are still many unanswered questions concerning mechanisms of TAD boundary formation and gene regulation. For example, the effect on gene expression upon deletion of CTCF-binding sites varies depending on their locations in the genome and cell types^40^. Enhancer and CTCF enrichment signals detected by ChIP-seq, distance between gene and regulatory elements, CTCF motif direction, and chromatin interaction frequency may be potential factors that can contribute to this variability, but there is not yet a clear formula that can predict the degree of effects. Other studies have shown that loss of cohesin eliminates most chromatin interaction loops but only a few genes near a subset of enhancers showed expression changes^41^. Clearly, further studies on the mechanisms that mediate chromatin structure formation and gene regulation are needed. Moreover, additional high-resolution chromatin contact maps are required to further investigate the chromatin interaction loops in other normal and prostate cancer subgroups. Although we have found that majority of genes that changed expression by TAD structure, epigenetic states, and loops do not have genetic alterations such as copy number variations and mutations (Supplementary Data 3, 4 and 9), higher resolution genotype data (e.g. whole-genome sequencing) may be needed to better understand the role of genetic alterations in chromatin structures.

In summary, we applied three different types of analyses to study mechanisms by which the prostate cancer transcriptome is controlled using 3D epigenomic maps. We provide a list of boundary-changed and epigenetic state-changed TADs, enhancer–promoter loops found specifically in prostate cancer cells, and a list of genes regulated by these mechanisms. The provided list will be a useful resource to the research community, which will enable a better understanding of prostate cancer biology and epigenomics.

## Methods

### Cell culture

The human prostate cancer C42B cells were obtained from ViroMed Laboratories (Minneapolis, MN, USA) whereas the human prostate cancer 22Rv1 (ATCC # CRL-2505) and normal prostate RWPE1 (ATCC # CRL-11609) cells were obtained from American Type Culture Collection (ATCC) (https://www.atcc.org/). Cells were grown at 37 °C in 5% CO_2_; the corresponding culture medium for each cell line (RPMI 1640 for C42B and 22Rv1 and Keratinocyte Serum Free Medium for RWPE1) was supplemented with 10% fetal bovine serum (Gibco by Thermo Fisher Scientific, Waltham, MA, USA) and 1% penicillin and streptomycin. All cell stocks were authenticated at the USC Norris Cancer Center cell culture facility by comparison to the ATCC and/or published genomic criteria for that specific cell line; all cells were documented as free of mycoplasma.

### In situ Hi-C

In situ Hi-C experiments were performed using RWPE1, C42B, and 22Rv1 cells following the original protocol by Rao et al.^5^ with minor modifications (such as the use of a 4-cutter restriction enzyme (MboI) instead of a 6-cutter restriction enzyme) as we have performed previously^13,40,42^. In situ Hi-C was performed in triplicate for C42B and in duplicate for 22Rv1 and RWPE1 (5 × 10^6^ cells were used for each experiment). One hundred units of *Mbo*I restriction enzyme (NEB, R0147) was used to digest the chromatin. For ligation, 2000U of T4 DNA Ligase (NEB, M0202) was added and samples were incubated at room temperature for 4 h with slow rotation. Hi-C chromatin was sheared to a size of 300–500 bp using a Covaris instrument (Covaris S2, Woburn, MA). Biotin-tagged DNA was pulled down using Dynabeads MyOne Streptavidin C1 beads (Life technologies, 65002) with 2× Binding Buffer (2× BB: 10 mM Tris-HCl (pH 7.5), 1 nM EDTA, 2 M NaCl). Each Hi-C library was amplified with 14 cycles of PCR, using Illumina primers. Each library was sequenced (75 bp paired-end) to produce ~500 M read pairs using an Illumina HiSeq 2000 per replicate (Supplementary Data 1). The quality of each Hi-C library was checked with FastQC^43^ and HiC-Pro version 2.8.0 (ref. ^44^). Raw fastq files were processed through the HiC-Pro pipeline version 2.8.0 (ref. ^44^) to create raw contact count matrices at multiple resolutions (e.g. 10, 20, 40, and 100 kb). The matrices were normalized using the iterative correction method (iced python library). After demonstrating that the individual Hi-C libraries were of high quality and reproducible, the Hi-C replicate datasets for a given cell line were pooled to increase the sequencing coverage. Hi-C data generated in this study are submitted to the NCBI GEO with accession number GSE118629.

### ChIP-seq

ChIP assays were performed in C42B, 22Rv1, and RWPE1 cells using H3K9me3 (Cat# 13969, Lot# 1; Cell Signaling and Technology, Inc.), H3K27me3 (Cat# 9733, Lot# 8; Cell Signaling and Technology, Inc.), and H3K36me3 (Cat# 2901, Lot# 3; Cell Signaling and Technology, Inc.) antibodies, according to the ENCODE standards (https://www.encodeproject.org/data-standards/). Each ChIP-seq experiment was performed in duplicate and ChIP-seq libraries were sequenced on an Illumina Hiseq machine. All ChIP-seq data were mapped to hg19 and peaks were called using MACS2 (ref. ^45^) after preprocessing data with the ENCODE3 ChIP-seq pipeline (https://www.encodeproject.org/chip-seq/). To call reproducible peaks from two replicates, the IDR tool (https://github.com/nboley/idr) for TF datasets or the naïve overlap tool for histone mark datasets was used, as suggested in the ENCODE3 ChIP-seq standards document (https://www.encodeproject.org/pages/pipelines/) and performed previously^13,46^. ChIP-seq data generated in this study are submitted to the NCBI GEO with accession number GSE118629. H3K27ac and CTCF ChIP-seq datasets, which are used to annotate active regulatory elements, are generated in previous studies^40^ and available from ENCODE (https://www.encodeproject.org/). FOXA1 ChIP-seq data in C42B^19^ were obtained from GSE40050 (Supplementary Data 1).

### Identification and classification of TADs

To identify TADs, the normalized Hi-C contact matrices, binned at 40 kb resolution, were used and domains were called using the TopDom program^14^; see Supplementary Data 2. The overlap analysis of TADs from three cell lines was performed using the following rules: if the boundaries of the domains are within 80 kb windows (2 blocks) or the domains are overlapped ≥ 80% between libraries, we considered that these TADs overlapped. Wilcoxon rank sum tests were performed to compare the size of TADs. Fisher’s exact tests were performed to compare the enrichment of TADs between groups. Raw *p* values are adjusted for multiple comparisons using the Benjamini and Hochberg method. The Vennerable R package (http://download.r-forge.r-project.org/src/contrib/Vennerable_3.0.tar.gz) was used to generate weighted Venn diagrams. Histone-mark-enriched TADs were defined as TADs that intersected with top 30k of ChIP-seq peaks (top 25%) and having most enriched average ChIP-seq signals (top 25%). Bedtools (https://github.com/arq5x/bedtools2) was used to intersect genomic regions and multiBigwigSummary function from deepTools^47^ was used to calculate average ChIP-seq signals per TAD using a fold change ChIP-seq bigwig file generated from MACS2 (ref. ^45^) using each histone-mark ChIP-seq vs input. To test whether TADs are functional units, we first chose H3K36me3-enriched TADs which has an average size of ~350 kb. We randomly generated 100 sets of TADs, which are in the range of 280 and 440 kb in the genome. Next, we counted how many TADs were enriched for H3K36me3 marks in randomly generated TADs to calculate empirical *p* value as we have done to call H3K36me3-enriched TADs. Gene desert TADs were defined as TADs that lack of genes (Gencode version 19, https://www.gencodegenes.org/human/release_19.html). Gene poor TADs were defined as TADs that have one to five genes per TAD. Gene-enriched TADs were defined as TADs that have more than five genes per TAD.

### Characterization of loops

Using the 10 kb resolution contact count normalized matrices, intra-chromosomal significant loops (using a 50 kb–10 Mb range) were selected for each cell line using the Fit-Hi-C program^15^ (*q*-value < 0.05). The anchors of the loops were intersected with active regulatory elements using a sequential classification scheme to sort the elements into promoter, defined as ±2kb windows of the TSS of all expressed genes (as determined using from RNA-seq data from the specific cell line), enhancer (top 25k H3K27ac peaks located greater than 2 kb from a known TSS of all genes identified using GENCODE), and insulator (among top 50k CTCF peaks, ones not overlapping with the sets of promoters or enhancers). The top 20 most frequent chromatin interaction categories are shown in Fig. 4a. To estimate the statistical significance of loop anchor overlap with the regulatory element, we randomly shuffled genomic regions 100 times to make artificial loop anchors (10 kb) in chromosome 1 and calculated the overlap percentage with the above regulatory elements. We found that real loop anchors in chromosome 1 had overlapped to most number of promoters, enhancers, and insulators (empirical *p* value < 0.01). Enhancer–promoter loops found from each cell line are listed in Supplementary Data 5. To identify statistically significantly differentially frequent enhancer–promoter loops between prostate cancer and normal cells, a Fisher’s exact test was performed between cancer and normal cell lines and adjusted *p* values accounting for multiple comparison issues (adj. *p* values < 0.05). Using the RCircos program^48^, Circos plots for FOXA1-binding sites and target genes of FOXA1-bound enhancers are generated (Fig. 5d) and the target genes are listed in Supplementary Data 8.

### RNA-seq

RNA-seq was performed in triplicate for RWPE1, C42B, and 22Rv1 cells. RNA was extracted using Trizol reagent (Cat # 15596-018; Thermo Fisher Scientific, NY, USA) and the quality of RNA was assessed using a 2100 Bioanalyzer instrument (Cat # G2939AA; Agilent Technologies), as we have performed previously^42^. After preparing cDNA using the SuperScript® VILO™ cDNA Synthesis Kit (Cat # 11754-050; Life technologies, Carlsbad, CA, USA), RNA-seq libraries were made using KAPA Stranded mRNA-Seq Kit with KAPA mRNA Capture Beads (Cat # KK8421; Kapa Biosystems, Woburn, MA, USA) and sequenced on an Illumina NextSeq 500 with 75 bp single end reads or on a HiSeq 3000 with 50 bp single end reads. Reads were mapped to hg19 GENCODE version 19 using STAR 2.4.1d^49^ and aligned reads were quantified at the gene level using Partek® Flow® software Quantify to annotation model (Partek Inc., St. Louis, MO, USA). The RPKM normalization method was used and log2 transformed. Genes expressed having a log2(RPKM + 1) > 1.5 on average from the triplicates were selected as actively expressed genes for active promoter identification. Differentially expressed genes between normal and cancer cells were selected by using the Gene Specific Algorithm from Partek® Flow® software using the upper quartile normalization method (Partek Inc., St. Louis, MO, USA) (adj. *p* value < 0.05) (Supplementary Data 7). Wilcoxon rank sum tests were performed to compare expression levels of genes in TADs and looped to enhancers. Raw *p* values are adjusted for multiple comparisons using the Benjamini and Hochberg method. All RNA-seq data were deposited in the NCBI GEO accession number, GSE118629.

### NOMe-seq

NOMe-seq was performed following the protocol we have reported in the previous study^18^. Chromatin was treated with the M.CviPI methyltransferase (Cat # M0227B; New England Bio Labs, Ipswich, MA), which methylates GpC dinucleotides that are not protected by nucleosomes or other proteins that are tightly bound to the chromatin. Following bisulfite treatment of the M.CviPI-methylated chromatin and subsequent genomic sequencing, we identified endogenous methylation (CpG) and accessibility (GpC) in the same sequencing reaction. The RWPE1 NOMe-seq library was sequenced using an Illumina HiSeq 2000 (100 bp paired-end), and NOMe-seq data were deposited in the NCBI GEO accession number, GSE118629; the C42B NOMe-seq library was previously generated^46^ (GSE102616). Each fastq file was aligned to a bisulfite-converted genome (hg19) using BSMAP^50^ and processed using the software programs Picard (http://picard.sourceforge.net) and SAMtools (http://samtools.sourceforge.net). To identify the methylation status of CpG sites (in all HCG trinucleotides) and GpC sites (in all GCH trinucleotides) from the bam file, the Bis-SNP^51^ program was used. For identification of NDRs, the find NDRs function in the aaRon R package (https://github.com/astatham/aaRon) was used with the *p* value cutoff 10^−8^. Top 50k of NDRs were used to find enhancer NDRs for motif analyses (Supplementary Data 6).

### Visualization of in situ Hi-C, ChIP-seq, and NOMe-seq data

In situ Hi-C chromatin interaction heatmaps were visualized using the HiTC R package^52^ at 10 kb resolution for Figs. 1–3, 6 and Supplementary Figs. 3 and 5. To visualize ChIP-seq and NOMe-seq data and generate genome browser screenshots, the Integrative Genomics Viewer (IGV)^53^ was used.

### Motif analysis

We used the findMotifsGenome.pl script from HOMER (http://homer.ucsd.edu/homer/)^54^ to discover known motifs at enhancer NDRs under enhancer–promoter loop anchors in normal (RWPE1) and tumor (C42B) prostate cells. The top 15 enriched motifs in these NDRs were identified by ranking motifs using *q*-values.

### Copy number estimates

To compare copy number estimates between prostate normal and cancer cells, we used the Genome-Wide Human SNP array (Mapping250K_Sty2) data in RWPE1 normal prostate and 22Rv1 prostate cancer cells^55^. Copy number data are processed using Aroma affymetrix R package^56,57^. We used the Genome-Wide Human SNP Array 6.0 data in 22Rv1 prostate cancer cells^58^ to identify genes whose expression levels are related to copy number changes (Supplementary Data 3, 4 and 9).

### The Cancer Genome Atlas analysis

RNA-seq data of prostate tissues (19 normal, 333 prostate adenocarcinoma) for The Cancer Genome Atlas (TCGA)^3^ was downloaded from the National Cancer Institute GDC Data Portal (https://portal.gdc.cancer.gov/). Normalized gene expression level of *FOXA1* gene (log2(RSEM + 1)) was used to generate a Supplementary Fig. 8. Student’s *t*-test was used to measure the gene expression difference between normal and tumor samples.

### Reporting summary

Further information on research design is available in the Nature Research Reporting Summarylinked to this article.

## Supplementary information

Supplementary Information

Description of Additional Supplementary Files

Supplementary Data 1

Supplementary Data 2

Supplementary Data 3

Supplementary Data 4

Supplementary Data 5

Supplementary Data 6

Supplementary Data 7

Supplementary Data 8

Supplementary Data 9

Reporting Summary

## Data Availability

The datasets generated during the current study are available in the NCBI Gene Expression Omnibus (GEO) under accession number GSE118629.
