# Supplementary material for: A high-resolution 3D epigenomic map reveals insights into the creation of the prostate cancer transcriptome
Source: Nat Commun. 2019 Sep 12;10:4154. doi: 10.1038/s41467-019-12079-8 (PMC6742760; doi:10.1038/s41467-019-12079-8)
Supplement: Supplementary file 1 — Supplementary Information [file 41467_2019_12079_MOESM1_ESM.pdf]

Supplementary Information for:

Rhie et al. “A high-resolution 3D epigenomic map reveals insights into the creation of the prostate cancer transcriptome”

Supplementary Figures

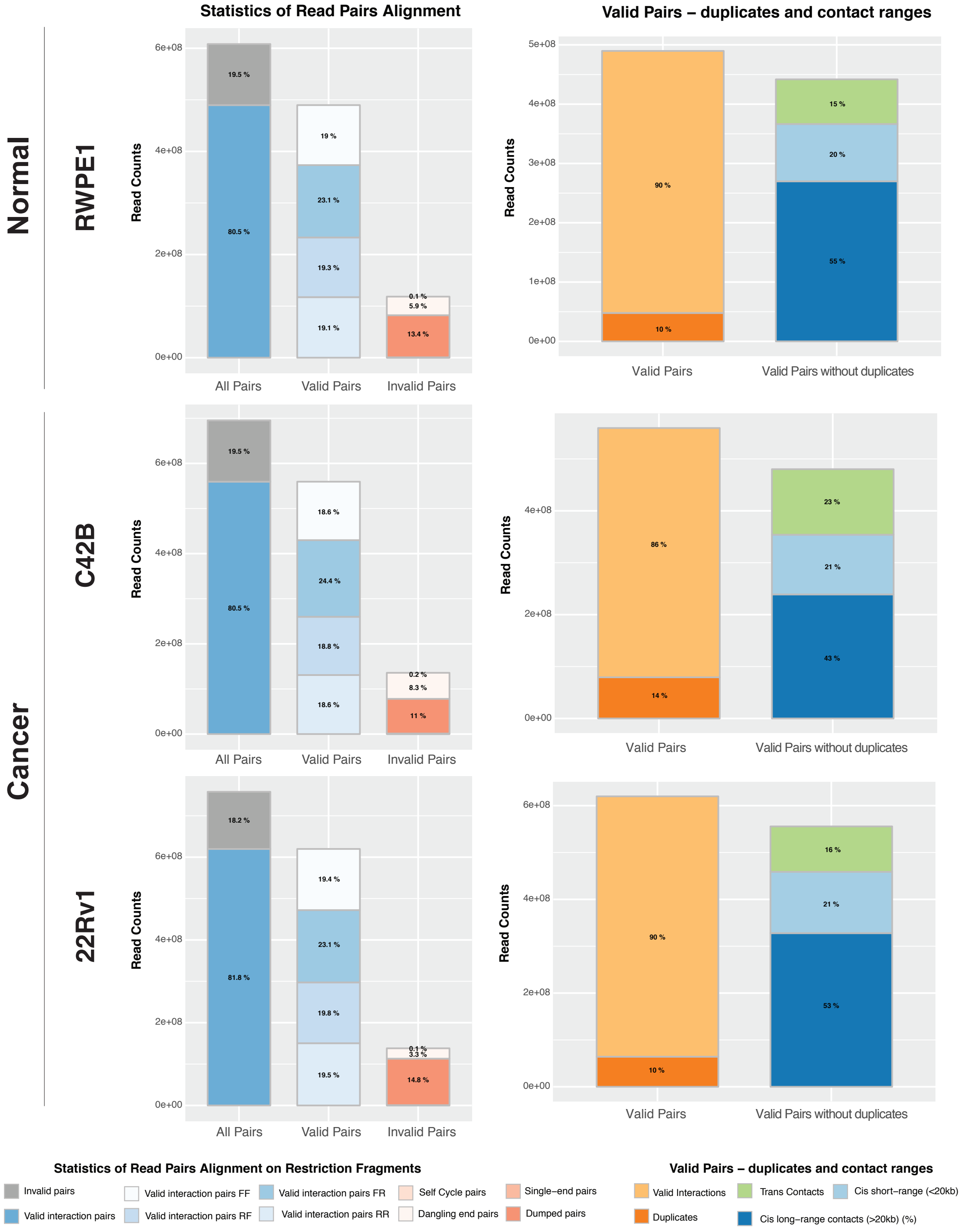

Supplementary Figure 1. Quality check of in situ Hi-C datasets. The left panel shows the fraction of different types of read pairs identified in each dataset; the majority of the read pairs were valid pairs. The right panel shows the number of unique valid pairs that are cis short-range, cis long-range and trans; most of the interactions are in cis (i.e. on the same chromosome).

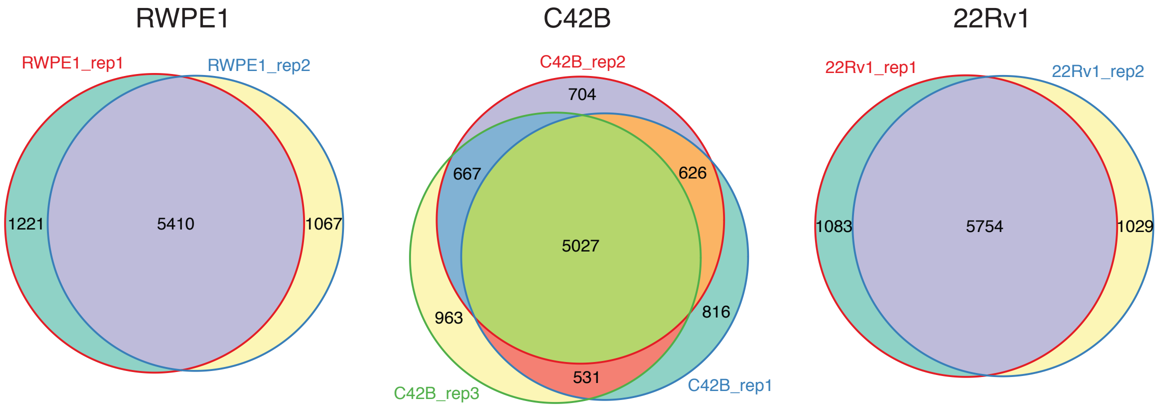

Supplementary Figure 2. TAD size comparison within cell lines. TADs called using RWPE1 (left), C42B (middle), and 22Rv1 (right) replicates are compared within cell lines.

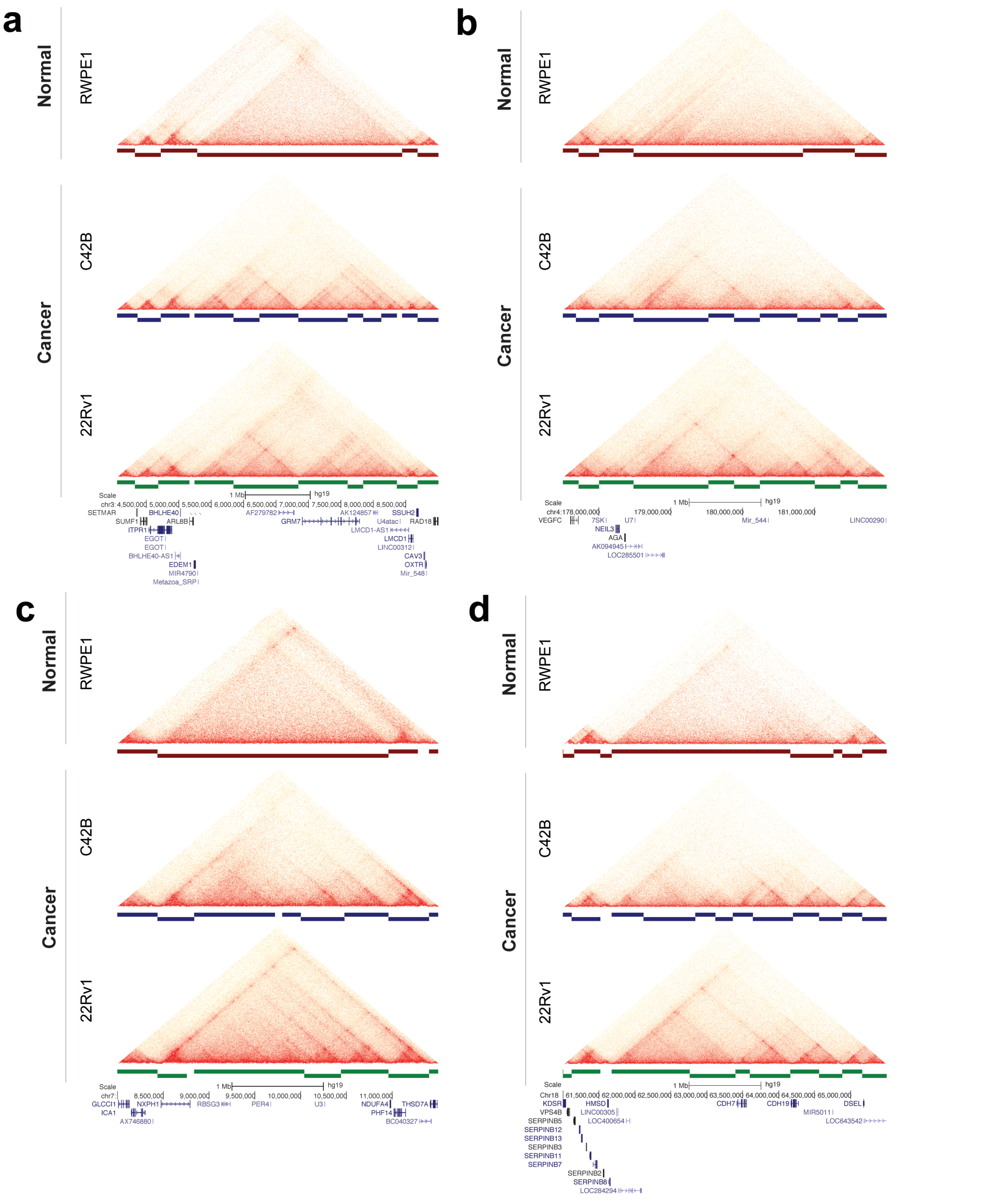

Supplementary Figure 3. Examples of TADs that are larger in normal cells compared to cancer cells. In situ Hi-C heatmaps and TADs in normal (RWPE1), cancer (C42B, 22Rv1) cells are shown for (**a**) chr3:4,050,000-9,000,000 (**b**) chr4:177,500,000-182,000,000 (**c**) chr7:8,000,000-11,500,000 and (**d**) chr18:61,000,000-65,500,000 region.

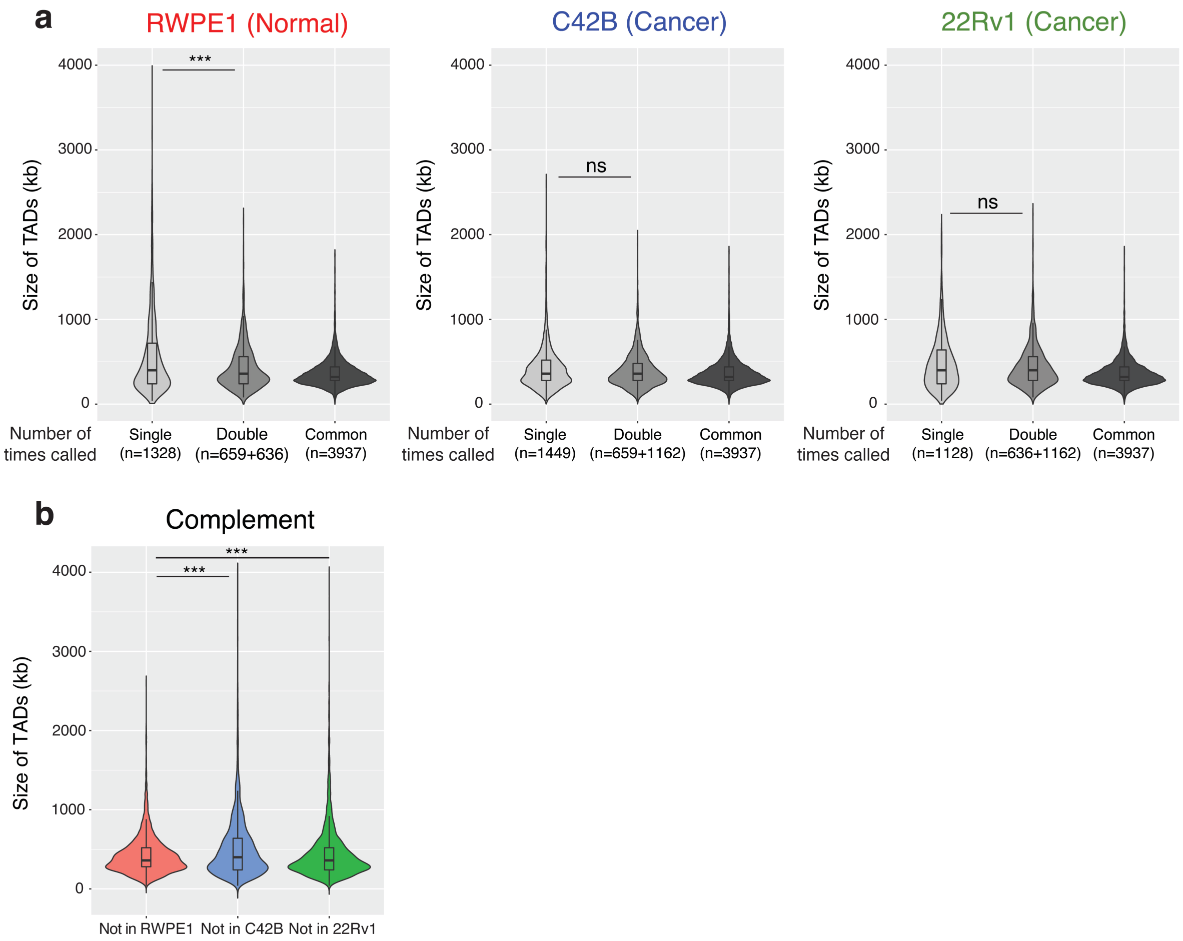

Supplementary Figure 4. TAD size comparisons between cell lines. (**a**) TAD size comparisons after stratifying TADs by the number of times overlapped, for RWPE1 (left), C42B (middle), and 22Rv1 (right). (**b**) TAD size comparisons for complement of TADs; red: all TADs except ones found in RWPE1 only, blue: all TADs except ones found in C42B only, green: all TADs except ones found in 22Rv1 only (Wilcoxon rank sum test, *adj. p-value <0.05, **adj. p-value <0.01, ***adj. p-value <0.001, ns (not significant) adj. p-value >0.05).

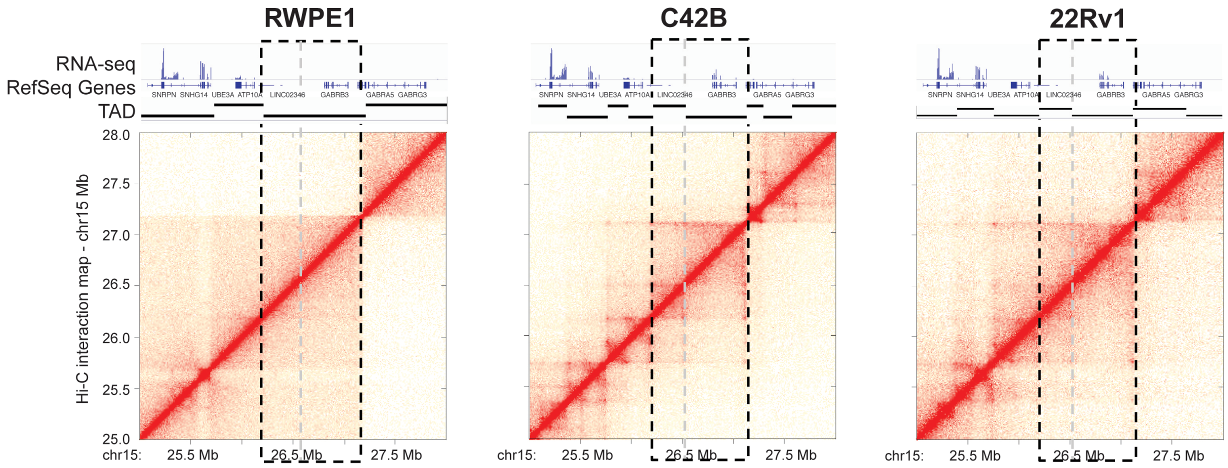

Supplementary Figure 5. An example of normal-specific and cancer-specific TAD. Shown is an example of normal-specific and cancer-specific TADs located in chromosome 15q12. RNA-seq tracks (top) and in situ Hi-C chromatin interaction maps (bottom) in normal (RWPE1) and prostate cancer (C42B, 22Rv1) are shown.

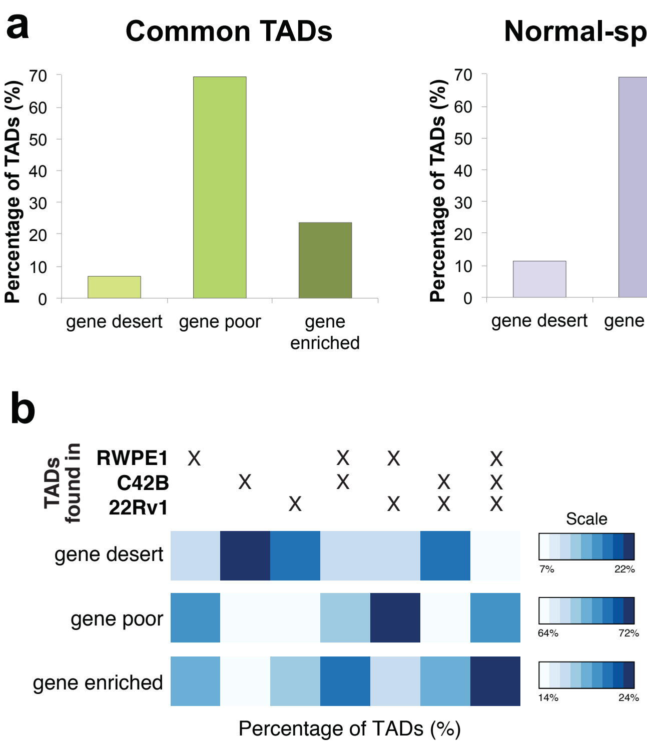

Supplementary Figure 6. Percentage of gene desert, poor and enriched TADs (**a**) Shown are bar graphs that reflect the distribution of gene desert, gene poor and gene enriched TADs for the common, normal-specific, and cancer-specific TADs. (**b**) For TADs found in one, two, and three cell lines are categorized by gene density (gene desert, gene poor, and gene enriched) and the percentage of TADs were plotted. Color scale: white - low percentage, dark blue - high percentage.

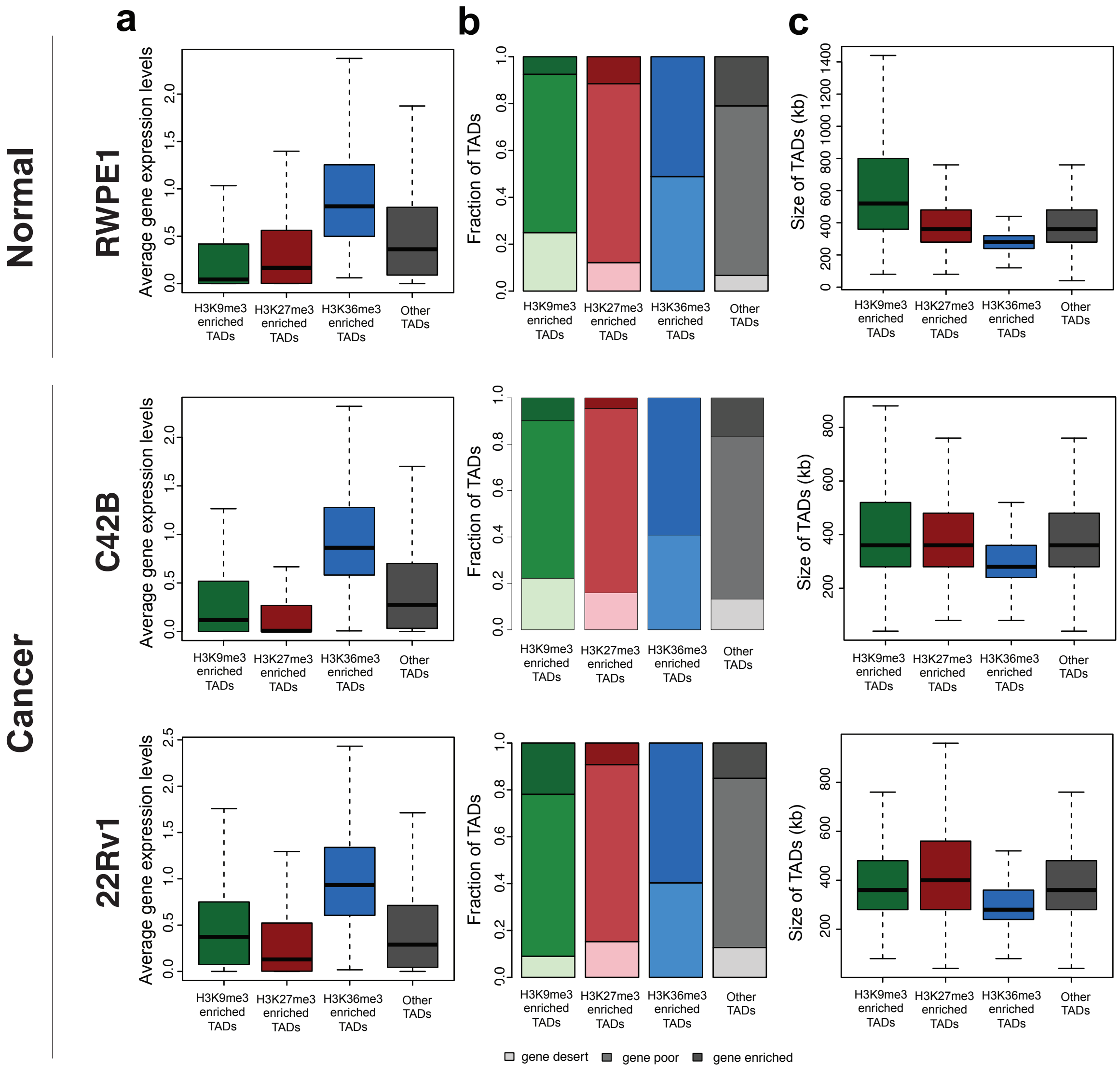

Supplementary Figure 7. Characterization of histone mark-enriched TAD subgroups. (**a**) Average expression levels of genes found in histone-mark enriched TADs and TADs without those histone marks (other). (**b**) Fraction of gene desert (light), gene poor (mid), and gene enriched (dark) TADs for the histone mark- enriched TAD subgroups and other TADs. (**c**) Shown is a size of histone mark-enriched TADs and other TADs. Top plots are for normal prostate (RWPE1) and bottom plots are for prostate cancer (C42B, 22Rv1).

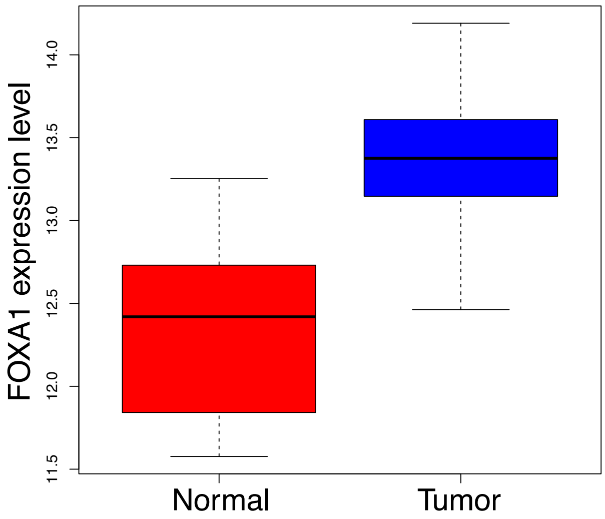

Supplementary Figure 8. FOXA1 expression level in normal and prostate tumor samples. FOXA1 is overexpressed in prostate tumor tissue samples (n=333) compared to normal prostate tissue samples (n=19) (Student's t test, p-value <4.24e-03). The Cancer Genome Atlas (TCGA) prostate adenocarcinoma gene expression datasets were used to generate this plot.
