# Supplementary material for: A high-resolution 3D epigenomic map reveals insights into the creation of the prostate cancer transcriptome
Source: Nat Commun. 2019 Sep 12;10:4154. doi: 10.1038/s41467-019-12079-8 (PMC6742760; doi:10.1038/s41467-019-12079-8)
Supplement: Supplementary file 2 — Description of Additional Supplementary Files [file 41467_2019_12079_MOESM2_ESM.pdf]

Description of Additional Supplementary Files

File Name: Supplementary Data 1
Description: Information about the next generation sequencing data used in this study.

File Name: Supplementary Data 2
Description: TADs identified by in situ Hi-C. (a) TADs identified in RWPE1 (b) TADs identified in C42B (c) TADs identified in 22Rv1 (d) Normal-specific TADs found in RWPE1 (e) Cancer-specific TADs found in C42B (f) Cancer-specific TADs found in 22Rv1 (g) Annotation of TADs in RWPE1 (h) Annotation of TADs in C42B (i) Annotation of TADs in 22Rv1.

File Name: Supplementary Data 3
Description: A list of upregulated genes from smaller size cancer-specific TADs in prostate cancer cells (a) C42B vs. RWPE1 and (b) 22Rv1 vs. RWPE1.

File Name: Supplementary Data 4
Description: A list of genes in epigenomic-switching TADs (a) between C42B and RWPE1 and (b) between 22Rv1 and RWPE1.

File Name: Supplementary Data 5
Description: Enhancer-promoter loops (a) identified in RWPE1 (b) C42B, and (c) 22Rv1 (d) Normal-specific enhancer-promoter loops (e) Cancer-specific enhancer-promoter loops (f) common enhancer-promoter loops.

File Name: Supplementary Data 6
Description: Nucleosome depleted regions found in (a) RWPE1 and (b) C42B.

File Name: Supplementary Data 7
Description: A list of genes differentially expressed (a) between C42B and RWPE1 and (b) between 22Rv1 and RWPE1.

File Name: Supplementary Data 8
Description: Target genes of FOXA1-bound enhancers.

File Name: Supplementary Data 9
Description: Overexpressed genes in prostate cancer cells with more frequent enhancerpromoter loops (a) C42B vs. RWPE1 and (b) 22Rv1 vs. RWPE1.
