# Supplementary material for: A high-resolution 3D epigenomic map reveals insights into the creation of the prostate cancer transcriptome
Source: Nat Commun. 2019 Sep 12;10:4154. doi: 10.1038/s41467-019-12079-8 (PMC6742760; doi:10.1038/s41467-019-12079-8)
Supplement: Supplementary file 12 — Reporting Summary [file 41467_2019_12079_MOESM12_ESM.pdf]

nature research

Corresponding author(s): Suhn Kyong Rhie, Peggy J Farnham

Last updated by author(s): Jun 20, 2019

Reporting Summary

Nature Research wishes to improve the reproducibility of the work that we publish. This form provides structure for consistency and transparency in reporting. For further information on Nature Research policies, see Authors & Referees and the Editorial Policy Checklist.

Statistics

For all statistical analyses, confirm that the following items are present in the figure legend, table legend, main text, or Methods section.

n/a	Confirmed	
☐	☒	The exact sample size (*n*) for each experimental group/condition, given as a discrete number and unit of measurement
☐	☒	A statement on whether measurements were taken from distinct samples or whether the same sample was measured repeatedly
☐	☒	The statistical test(s) used AND whether they are one- or two-sided<br>*Only common tests should be described solely by name; describe more complex techniques in the Methods section.*
☐	☒	A description of all covariates tested
☐	☒	A description of any assumptions or corrections, such as tests of normality and adjustment for multiple comparisons
☐	☒	A full description of the statistical parameters including central tendency (e.g. means) or other basic estimates (e.g. regression coefficient) AND variation (e.g. standard deviation) or associated estimates of uncertainty (e.g. confidence intervals)
☐	☒	For null hypothesis testing, the test statistic (e.g. *F*, *t*, *r*) with confidence intervals, effect sizes, degrees of freedom and *P* value noted<br>*Give P values as exact values whenever suitable.*
☒	☐	For Bayesian analysis, information on the choice of priors and Markov chain Monte Carlo settings
☒	☐	For hierarchical and complex designs, identification of the appropriate level for tests and full reporting of outcomes
☒	☐	Estimates of effect sizes (e.g. Cohen's *d*, Pearson's *r*), indicating how they were calculated

Our web collection on statistics for biologists contains articles on many of the points above.

Software and code

Policy information about availability of computer code

Data collection	SRA Toolkit
Data analysis	FastQC, HiC-Pro version, Fit-HiC, Bwa, Picard, MACS2, IDR, TopDom, Bedtools, Vennerable, RCircos, IGV, Partek Flow

For manuscripts utilizing custom algorithms or software that are central to the research but not yet described in published literature, software must be made available to editors/reviewers. We strongly encourage code deposition in a community repository (e.g. GitHub). See the Nature Research guidelines for submitting code & software for further information.

Data

Policy information about availability of data

All manuscripts must include a data availability statement. This statement should provide the following information, where applicable:

- Accession codes, unique identifiers, or web links for publicly available datasets
- A list of figures that have associated raw data
- A description of any restrictions on data availability

All in-house generated sequencing data were deposited in the NCBI GEO accession number, GSE118629

Field-specific reporting

Please select the one below that is the best fit for your research. If you are not sure, read the appropriate sections before making your selection.

☒ Life sciences ☐ Behavioural & social sciences ☐ Ecological, evolutionary & environmental sciences

For a reference copy of the document with all sections, see nature.com/documents/nr-reporting-summary-flat.pdf

October 2018

Life sciences study design

All studies must disclose on these points even when the disclosure is negative.

Sample size	Normal prostate (RWPE1) and prostate cancer (C42B and 22Rv1) cells were used. More than two replicates of in situ Hi-C, ChIP-seq, and RNA-seq data were generated.
Data exclusions	No data were excluded from the analyses.
Replication	All attempts at replication were successful.
Randomization	This study did not use animals and human participants. It is not relevant to this study.
Blinding	This study did not use animals and human participants. It is not relevant to this study.

Reporting for specific materials, systems and methods

We require information from authors about some types of materials, experimental systems and methods used in many studies. Here, indicate whether each material, system or method listed is relevant to your study. If you are not sure if a list item applies to your research, read the appropriate section before selecting a response.

Materials & experimental systems

n/a	Involved in the study	
☐	☒	Antibodies
☐	☒	Eukaryotic cell lines
☒	☐	Palaeontology
☒	☐	Animals and other organisms
☒	☐	Human research participants
☒	☐	Clinical data

Methods

n/a	Involved in the study	
☐	☒	ChIP-seq
☒	☐	Flow cytometry
☒	☐	MRI-based neuroimaging

Antibodies

Antibodies used	ChIP assays were performed in C42B, 22Rv1 and RWPE1 cells using H3K9me3 (Cat# 13969 Lot# 1, Cell Signaling and Technology, Inc.), H3K27me3 (Cat# 9733 Lot# 8, Cell Signaling and Technology, Inc.), and H3K36me3 (Cat# 2901 Lot# 3, Cell Signaling and Technology, Inc.) antibodies.
Validation	According to ENCODE standards (https://www.encodeproject.org/data-standards/), antibodies were validated.

Eukaryotic cell lines

Policy information about cell lines

Cell line source(s)	The human prostate cancer C42B cells were obtained from ViroMed Laboratories (Minneapolis, MN, USA) whereas the human prostate cancer 22Rv1 (ATCC # CRL-2505) and normal prostate RWPE1 (ATCC # CRL-11609) cells were obtained from ATCC (https://www.atcc.org/).
Authentication	All cell stocks were authenticated at the USC Norris Cancer Center cell culture facility by comparison to the ATCC and/or published genomic criteria for that specific cell line.
Mycoplasma contamination	All cells were documented as free of mycoplasma.
Commonly misidentified lines (See ICLAC register)	n/a

ChIP-seq

Data deposition

☒ Confirm that both raw and final processed data have been deposited in a public database such as GEO.

☒ Confirm that you have deposited or provided access to graph files (e.g. BED files) for the called peaks.

Data access links *May remain private before publication.*	https://www.ncbi.nlm.nih.gov/geo/query/acc.cgi?acc=GSE118629

Files in database submission	In situ Hi-C, ChIP-seq, RNA-seq, and NOMe-seq raw and processed files are deposited to GEO; please see Table S1.
Genome browser session (e.g. UCSC)	n/a

Methodology

Replicates	RWPE1 H3K9me3 ChIP-seq rep1 and 2, RWPE1 H3K27me3 ChIP-seq rep1 and 2, RWPE1 H3K36me3 ChIP-seq rep1 and 2, C42B H3K9me3 ChIP-seq rep1 and 2, C42B H3K27me3 ChIP-seq rep1 and 2, C42B H3K36me3 ChIP-seq rep1 and 2, 22Rv1 H3K9me3 ChIP-seq rep1 and 2, 22Rv1 H3K27me3 ChIP-seq rep1 and 2, 22Rv1 H3K36me3 ChIP-seq rep1 and 2
Sequencing depth	According to ENCODE3 ChIP-seq pipeline, more than 40M unique reads were sequenced per replicate.
Antibodies	ChIP assays were performed in C42B, 22Rv1 and RWPE1 cells using H3K9me3 (Cat# 13969 Lot# 1, Cell Signaling and Technology, Inc.), H3K27me3 (Cat# 9733 Lot# 8, Cell Signaling and Technology, Inc.), and H3K36me3 (Cat# 2901 Lot# 3, Cell Signaling and Technology, Inc.) antibodies.
Peak calling parameters	All ChIP-seq data were mapped to hg19 and peaks were called using MACS2 after preprocessing data with the ENCODE3 ChIP-seq pipeline (https://www.encodeproject.org/chip-seq/). To call reproducible peaks from two replicates, the IDR tool (https://github.com/nboley/idr) for TF datasets or the naïve overlap tool for histone mark datasets was used, as suggested in the ENCODE3 ChIP-seq standards document (https://www.encodeproject.org/pages/pipelines/) and previously described.
Data quality	Library complexity and ChIP quality were assessed using ENCODE quality metrics and standards (e.g. NRF, PBC, NSC, RSC). To call reproducible peaks from two replicates, the IDR tool (https://github.com/nboley/idr) for TF datasets or the naïve overlap tool for histone mark datasets was used, as suggested in the ENCODE3 ChIP-seq standards document (https://www.encodeproject.org/pages/pipelines/) and previously described.
Software	BWA, Picard, MACS2, IDR
